# Report of the Committee on Medical Education

**Published:** 1854-03

**Authors:** 


### ART. III. — *Report of the Committee on Medical Education.*

This report, embodied in the Transactions of the National Medical Association, is from the pen of the chairman of the committee, Dr. Z. Pitcher, of Detroit. Thrown out to the profession under the sanction of the highest medical authority in this country, it deserves from its own ability and intrinsic merit, as well as from the medium through which it is circulated, a careful consideration.

The subject of medical education is one which lies at the foundation of the honor of the profession. A false step in this beginning of medical life, the bias or feeling which the young physician may imbibe from his alma mater, will give tone to his subsequent conduct. His ideas of perfection in practice will be derived from the relative perfection of his teachers; the graduate of an inferior school will have lower ideas of professional excellence, than he, however unqualified himself, who goes out to the world from an institution of high character and tone. So true is this, that it is rare to see a mind, however shrewd and astute it may be naturally, recover from the negative results of poor teaching, and accomplish for itself, in the fields of practice, that task of improvement to which its student years should have been devoted. He who graduates high in a superior school, retains through life he superiority with which he started.

The American Journal of the Medical Sciences for January, in reviewing in detail the Transactions of the National Association, begins its notice of Dr. Pitcher's report in the following terms:

"It is marked throughout by a manly independence of thought on the important subjects involved in the general subject of which it treats, and much sound sense in the conclusions to which its author has arrived. It may, perhaps, disappoint, on the one hand, such as had formed for themselves an ideal model of a perfect system of medical education, which, without the slightest consideration as to the peculiar characteristics of our national character and institutions, and the state of society throughout a large portion of our widely extended territory, they had hoped to see speedily carried into effect, through the instrumentality of the American Medical Association; and, on the other hand, induce those who look upon the present condition of our leading medical schools as demanding little, if any, improvement, to exult in the supposed abandonment, by the Association, of the high ground assumed by it, in its outset, in respect to medical education. By a careful perusal of the report, however, it will be perceived, we think, to be as positive in maintaining the necessity of arousing 'the profession of the country to such an effort as shall shake from it the reproach of ignorance and of quackery, by which it has been humbled in its own estimation, and abased in that of its fellow-men,' as either of its predecessors."

While we cheerfully concede honesty of purpose, manly independence of thought, and true devotion to the interests of the profession, to this report, we are unwilling to accede to its doctrines, or join in the laudations of the American Journal of Medical Sciences, so far as they relate to the organization of medical schools in this country. Without any disposition "to exult in the supposed abandonment, by the Association, of the high ground assumed by it, in its outset, on the subject of medical education," and with a sincere regret if there has been any such abandonment, we are one of that numerous class, "who look upon the present condition of our leading medical schools, as demanding little, if any improvement," at the hands of government, or in the manner proposed by Dr. Pitcher. Indeed, we look upon his proposed improvements as a progression backward, a lowering of the standard of requirement, and a most decided injury to the profession at large.

We have already distinctly expressed our views upon the subject of government schools. We have, as we think, shown upon a previous occasion, that the success and efficiency of a school depended upon the motives acting upon its teachers; that private interest and reputation, stimulated by free competition, would be far more likely to make a good school, than would the soporific influence of a fixed salary from government, regardless of success or efficiency.

We think we see, in the report of Dr. Pitcher, the result of that gentleman's propinquity, and *quasi* connection with the only government school in the country. Naturally these causes, together with the large influence Dr. P. has had in the organization of the school, and the warm interest he feels in its success, would, in some measure, bias his opinions. We only wonder that so strong a thinker, and so able a writer, has not made out a better case for his favorite theory.

The author, after alluding to the number of shrewd quacks found scattered through the country as a serious evil, suggests "the establishment of free colleges for the preparatory and professional education of the young men now scattered over the wide and half-cultivated domain of the West," as a remedy.

It is our task to inquire whether or no this would be a remedy, and to examine the affirmative arguments of the report.

"To show that such a measure can be carried into effect, notwithstanding the popular character of our political institutions," the report gives a succinct account of the organization of the University of Michigan. While we admit the theoretical excellence of this organization, and defer any objections to the tedium of a seven months' course, or the false policy of deducting a year from the term of study of those who have received a competent literary education, we are unwilling to acknowledge that the result hoped for has been obtained. All the merits of this school are easily attainable by other colleges; its demerits are inherent in it; it bears the constitutional taint of political influence in its appointments; of lack of clinical teaching; and, more than all, a method of paying its teachers which insures laziness, and avoids all responsibility, save to the political party in power. And nothing but the competitional system can restore to it, or rather confer upon it, any health, or vigor.

It seems to have been necessary, in this report, to ignore, in some manner, the value of clinical instruction. We have demonstrated in a previous article, upon a kindred subject, that no success, or efficiency, has attended medical teaching in Europe, or America, except it afforded ample opportunities for the study of disease, in its presence. This difficulty is overcome in the report, by the statement of the question in the following language:

"We believe that this subject should be examined from two distinct points of view, one of which exhibits the character and qualifications of those who graduate in the best schools, and the other the character and influence in society of that other multitude, who choose to exclude themselves from these advantages, but come, nevertheless, by some illegitimate entrance, into the great professional amphitheater."

The report argues that the first class do not derive any essential benefit from clinical instruction; that there is not a sufficient concordance between the lectures of the college, and the clinic at the hospital; that the sight of fistula in ano, and lues venerea, does not tend to elucidate a lecture just listened to on pleuro-pneumonia, and that the wards are crowded, and students see but little.

Were we to admit that these statements were exact — though we believe that they will apply to Philadelphia alone — we can see in them only a necessity of reforming clinical teaching. If it is not doing all the good of which it is capable, then let us make it more efficient, and not throw a good thing away because it is badly managed.

But of the second class of students — those lost sheep of Israel, "who choose to exclude themselves from these advantages." The argument is briefly this, and we trust our readers will pardon our abridgment of it:

They are cheap men — build them cheap schools. They will not pay for an education — ergo, give it them for nothing. They are first-rate scamps out of the profession — bring them among us, and they will be only second-rate scamps. The aggregate character of the profession will be lowered thereby — but quackery will be correspondingly enfeebled. People do not appreciate good physicians — give them, therefore, poor ones.

The author of the report quotes John Bell to show the futility of clinical teaching, until the student has arrived at a knowledge of anatomy. While we can see the advantages to be derived from such a knowledge, and can appreciate its necessity, we do not see as clearly, that clinical teaching is so worthless, even to the tyro in medicine.

Much of the success of the auscultator depends, not only on his quickness and erudite hearing of the sounds, but upon his method of manipulation in educing the sounds. Clinical teaching finds its highest good, not in any theoretical knowledge it confers, but in the numberless little things, which the most unobservant student must see, and which, in the aggregate, go far to make the skillful and *resourceful* physician. He learns the manner of holding a knife, of introducing a catheter, of the practice of physical diagnosis; he becomes familiar with the uses of the endless mechanical contrivances which so increase the usefulness of practical medicine; and becomes familiar, if not with the pathology, at least with the countenance of disease. He acquires firmness in danger, quickness in apprehension, readiness in adapting means to ends. In a word, he has practice, and it goes far to make him a practical man.

A student who had applied the stethoscope to the epigastric region, to hear a bellows murmur of the heart, much to the amusement of older men, complained to us, as we left the hospital, that it was all fighting in the dark to him. He saw many things done which he could not understand; much of the lecture itself was unintelligible; he thought he was not far advanced enough in his studies to profit by the clinics.

But upon questioning him, we found that he had learned that morning, many negative, and some positive truths, which he was hardly conscious of himself. He had learned what was the hippocratic countenance; he had learned that patients sometimes die under the best of treatment; he thought he could dress a fracture of the tibia himself; and he was very sure he should never forget the region of the heart, or the sound of the bellows murmur.

It seems a work of supererogation to defend clinical teaching, but this report seriously underrates its advantages, and the American Journal of Medical Sciences "fully coincides" in its remarks "on the total inefficiency and absurdity of the system of clinical instruction, as now generally pursued in the hospitals of the United States." We believe that we are not alone—more—that the intelligent sentiment of a vast majority of the profession is with us, in conceding to clinical teaching the first rank in the means of the student's advancement.

If more colleges, and free colleges, are necessary; if they must be organized in out-of-the-way places, to catch out-of-the-way men, and where clinical teaching is impossible, why not state the question fairly, and say, "we offer to you an inferior school, but it costs nothing." The attempt to place the diploma of such schools upon an equality with schools of high advantages; to place state monopolies in competition with institutions depending on private enterprise and excellence; and to cast out into the world of practice a class of men who have studied medicine because its study cost them nothing, to compete with men who have given their time, money, and sacrifices, that they might enter an honorable profession, not as charity boys, but as men who had earned their foothold in it, requires better arguments in its favor, than any we have yet seen.

We notice this report thus formally, because it comes from a high source; it bears the apparent approval of the highest medical organization in our country; its author is a man of age and talent, who has earned a right to a careful consideration of any views he may promulgate, by a long and distinguished career of honorable effort; and, finally, because it bears the endorsement—hasty and unconsidered we hope—of the great name of the American Journal of Medical Sciences.

In closing, we may say that, aside from the positions we except to, this report is characterized by ability. In all those parts of his report where his home associations do not bias it, the author maintains nobly a high ground, and enforces it by sound and logical argument. He seems, in urging the necessity of preparatory education, and the benefit which classical and literary knowledge must confer upon the medical thinker, to feel more at home than in depreciating clinical instruction. His sentences have an easier flow, and he shows a consciousness, that here at least, he was upon a high and noble theme. H.

